# Bacterial Virus Lambda Gpd-Fusions to Cathelicidins, α- and β-Defensins, and Disease-Specific Epitopes Evaluated for Antimicrobial Toxicity and Ability to Support Phage Display

**DOI:** 10.3390/v11090869

**Published:** 2019-09-17

**Authors:** Sidney Hayes

**Affiliations:** Department of Biochemistry, Microbiology & Immunology, College of Medicine, University of Saskatchewan, Saskatoon, SK S7N 5E5, Canada; sidney.hayes@usask.ca; Tel.: +1-306-966-4307

**Keywords:** bacterial virus, bacteriophage (phage) lambda, gpD gene-fusion, capsid display, human α-defensins, HNP1, HD5, human β-defensins, HBD3, DEFB126, cathelicidins, human LL37, porcine PR39, capsid display density, single epitope particle vaccines

## Abstract

We showed that antimicrobial polypeptides, when translated as gene fusions to the bacteriophage lambda capsid decoration protein gpD, formed highly toxic molecules within *E. coli*, suggesting that they can retain their antimicrobial activity conformation when fused to gpD. These include gpD-fusions to human and porcine cathelicidins LL37 and PR39, β-defensins HBD3 and DEFB126-Δ (deleted for its many COOH-terminal glycosylation sites), and α-defensin HD5. Antimicrobial toxicity was only observed when the peptides were displayed from the COOH-terminal, and not the NH_2_-terminal end, of gpD. This suggests that COOH-terminal displayed polypeptides of gpD-fusions can more readily form an active-state conformation than when they are displayed from the NH_2_-terminal end of gpD. The high toxicity of the COOH-displayed gpD-defensins suggests either that the fused defensin peptides can be oxidized, forming three correct intramolecular disulfide bonds within the cytosol of bacterial cells, or that the versions without disulfide bonds are highly toxigenic. We showed the high efficiency of displaying single epitope 17 amino-acid fusions to gpD on LDP (lambda display particles), even when the gpD-fusion protein was toxic. The efficient formation of high display density LDP, displaying a single disease specific epitope (DSE), suggests the utility of LDP-DSE constructs for use as single epitope vaccines (SEV).

## 1. Introduction

**Figure 1:**
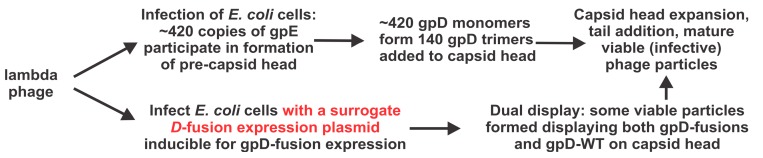
Requirement and participation of gpD wild type (gpD-WT) and/or gpD-fusions in formation of viable lambda phage particles.

The gpD capsid decoration protein of lambda is essential for the formation of viable phage particles, Figure 1. Each capsid consists of about 405 to 420 copies [1]. The model is that during phage morphogenesis and DNA packaging into the preformed phage prohead, comprising mainly gpE, there is an expansion event which exposes new sites on gpE, enabling the binding of gpD to form gpD-trimers that stabilize the enlarged capsid head [2,3]. gpD only becomes redundant if the phage genome is lower than 82% of the wild type lambda genome [4,5]. gpD is an essential protein for all the variants of lambda used herein.

All phage display strategies involve fusing the coding region for a protein, polypeptide(s) or peptide(s) to a phage surface capsid gene. The capsid-fusion gene is expressed from a phage genome engineered to include the gene fusion or is expressed from a surrogate genome. When the gene fusion is inserted within the intact viral genome, all formed virus particles will potentially be coated with a capsid-polypeptide fusion protein. The success of this recombination procedure depends on whether the capsid fusion protein can be added to the capsid and if the modified viral particle is infective. Recombinant phage display strategies used with the lambda genome, and involving fusions to one or more of the phage lambda capsid genes, have permitted the preparation of lambda recombinants that display a peptide capable of binding to a unique ligand [4,6,7,8,9,10,11,12,13,14,15,16]. Early studies showed that fusions could be made to the NH_2_- or COOH-termini of gpD, and that proteins as large as β-lactamase and tetrameric β-galactosidase were displayed in a functionally active form, at a density of 34 copies of β-galactosidase per capsid [11]. In a systematic study, Gupta et al., [17] examined the display density of pre-selected plaque-forming lambda-recombinant phages, each engineered to encode a *D*-fusion gene. The lambda capsid head in their recombinant system could efficiently display *D*-fusion additions of 72, 156 or 231 amino acids (AA). Not all recombinants encoding gpD-fusions can generate pfu. Some problematic gpD-fusion polypeptides are recognized as being “recalcitrant” and obviously prevent capsid formation and the generation of infectious phage particles [18,19].

**Figure 2:**
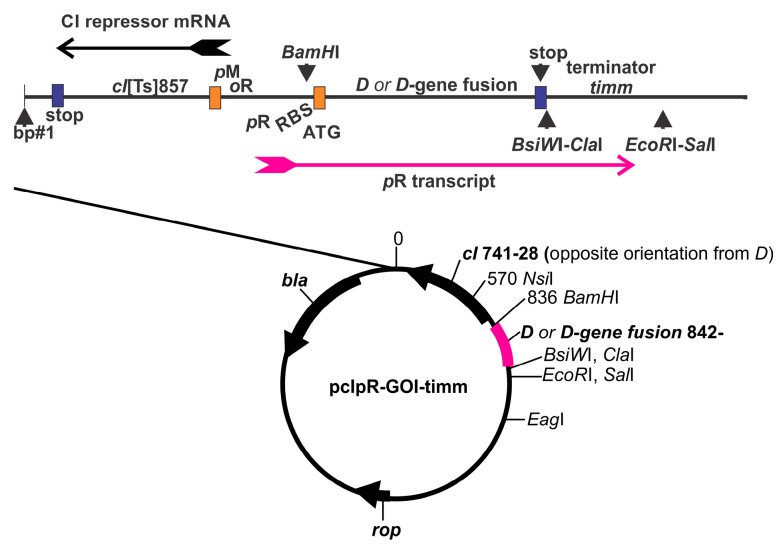
Map for the synthetic plasmid pcIpR-GOI-timm expression vector, used to clone any Gene of Interest (pink), e.g., the *D-*fusion gene shown, with ATG for GOI at base 842 of plasmid. In the versions of this plasmid used herein where the gene of interest (GOI) represents *D* or *D-*fusions, the wild type sequence for *D* was synthetically optimized in 55 of its 110 codons [26,27] to improve translation efficiency. This version of *D* is termed *D*coe and the protein product is called gpDcoe (representing *co*don *e*nhanced). The sequence of *D*coe is provided in the supplement to reference [26], where *D*coe was fused to a linker and four regions of the porcine Circovirus 2 capsid gene, i.e., on plasmid pcIpR-*D*-CAP-timm. Base assignments after 842 depend on the size of the GOI insertion and so are not shown. All versions of the pcIpR-GOP-timm expression plasmid [26,28,29,30,31] include the strong rightward lambda promoter *pR*. The expression of a *D* or *D*-fusion gene cloned as GOI into pcIpR-GOI-timm is negatively regulated.

An alternative strategy for phage display, other than producing a genomic recombinant, is to express a capsid fusion gene from surrogate genome (e.g., expression plasmid, Figure 2). The fusion gene, either purified, or as part of a cell extract, can be added to a virus particle *intermediate* in an in vitro phage morphogenesis system [20,21,22,23,24,25]. Depending on the arrangement, the fusion protein may or not compete with the natural capsid protein for addition to the capsid. In another version, *dual* capsid proteins [18,26] can be formed in vivo when a cell expressing a plasmid-encoded capsid fusion gene is infected with a virus encoding a wild type capsid protein. Mosaic bacterial virus display particles, Figure S1, in theory, can display both the wild type capsid and the capsid-fusion protein. This can potentially circumvent situations where the capsid fusion protein, in the absence of any wild type capsid protein, is incompatible with the assembly of stable phage particles. The efficiency (display density achieved) for the addition of a gpD-fusion to the capsid using the surrogate (dual) genome expression system varies when cells are infected with *D*^+^ phage. The gpD-fusion becomes the sole capsid addition possibility when surrogate expression cells are infected with phage defective in *D*.

There are no rules for what will work well in lambda phage display. Gupta et al. [17] found that small peptides can be displayed efficiently from recombinant phage, but much of the literature suggests that nearly any fusion combination can produce some level of capsid display. We used the dual display lambda technology to coat lambda display particles (LDP), fusing gpD to polypeptides arranged as string-of-beads multiple disease specific epitopes (DSE) to make LDP-DSE vaccines, which, when administered to animals each elicited an immune response to the DSE [27,28]. We had empirically noted that different gpD-DSE individually influence in vivo LDP-DSE morphogenesis/particle formation and display density. Accordingly, we examined the influence of gpD-fusion toxicity and their ability to complement LDP formation. *D*-fusions were created that included a six amino acid linker sequence joined to (rather short) antimicrobial peptides/polypeptides ranging in size from 35 to 85 amino acids (AA), or to a single DSE epitope of 11 AA. Antimicrobial peptides (AMPs) were chosen for gene fusions because it was anticipated that they would add an easily assayable activity (bacterial toxicity), if they could form biologically active conformations when fused to gpD.

## 2. Materials and Methods 

### 2.1. Strains 

The *E. coli* strain 594 [29] was transformed with pcIpR-GOI-timm variants, Figure 2, which confer ampicillin resistance and *imm*λ repression at 30 °C. Phages λ*cI*857*D*am123 and λ*imm*434*D*am123 were obtained from Dr. Lynn Thomason, NCI, Bethesda and we sequenced the *D*am123 mutation to λ base pair 6029, changing codon 95 of *D* from GAG to TAG. The *D*am123 phages efficiently form plaques on cell lawns of the permissive *E. coli* strain TC600 *supE* which encodes an amber suppressor tRNA. Only *D* amber revertants of *D* amber 123 phages form plaques on the non-permissive 594 host. The preparations of λ*imm*434*D*am123 employed were exclusively grown up on TC600 cells (see Section 2.7.1 and Section 2.7.2).

### 2.2. Growth Medium and General Dilution “Buffer” 

LB-Amp50 medium (10 g Bacto Tryptone, 5 g Bacto yeast extract 5 g NaCl/L) with 50 µg/mL Ampicillin. “Buffer” used without any additional descriptors is 0.01 M Tris, 0.1 M NaCl, pH 7.6.

### 2.3. Plasmid Construction; D-Fusion Plasmids and Encoded Amino Acids

All the plasmids employed were made by inserting intact genomic blocks (gBlocks, Integrated DNA Technologies, Coraville, IA) designed to represent versions of gene *D* or *D*-fusions between the *BamH*I and *Cla*I restriction sites in plasmid pcIpR-GOI-timm (Figure 2). Cloned plasmid possibilities for each construct were then transformed into strain 594. Four or five of the colony-isolated transformants were selected for plasmid sequencing analysis and those verified as having the correct *D* or *D*-fusion sequence were frozen back and used for bioassays. The plasmid strains employed are described in Table 1. The “pXXX” number represents the pcIpR-Dcoe-fusion-timm plasmid construction/strain number. Plasmid Dcoe^-^ (p613*) includes a 67 bp in frame deletion in the *D*coe sequence present in p613. The codons for the NH_2_-terminal or COOH- terminal additions to *D* were optimized for translation in *E. coli.* LL37 [32,33,34,35] and PR39 [36] are human and porcine cathelicidins; the designations HNP1 [37] and HD5 designate α-defensins [38], while HBD3 [38] and DEFB126 [39,40] are β-defensins.

### 2.4. Single-Burst LDP-Vaccine and SEV Production 

The routine procedure for obtaining high titer phage lysates involves a two-burst system, where cells are infected at a low multiplicity of infection (MOI), e.g., ~0.1, phage added per cell, as described in Figure 5 of [26]. When the first round of infection and cell burst occurs, the released phage should have increased sufficiently in titer to infect all the remaining uninfected cells in the growing culture, i.e., with MOI >1.0. A high titer lysate was obtained following the second round of infection. This procedure is not ideal for preparing phage display particles coated by expression of a *D*-fusion gene encoded on a plasmid: All the phage display particles released from the first infection that subsequently bind to and infect cells to initiate the second round of phage burst are lost, because they do not encode the gene fusion. Therefore, a single-burst phage display procedure, e.g., with an initial MOI of 3 to 5, is preferable for preparing LDP-vaccines (described in Supplementary File S1). 

### 2.5. Infection-Complementation Using Transformed Cells

This information was described in [26,27,28,29,30,31]. Cells transformed with the plasmid pcIpR-GOI-timm, Figure 2, encode the *imm*λ CI[Ts857] repressor which establishes an immune state so that infection by *imm*λ is blocked when the cells are grown at below ~40–42 °C. Nevertheless, it is possible to complement for activity of the GOI between ~36–37 °C through 42 °C from this plasmid [29,31]. Thus, the GOI is expressible at lower temperatures than *imm*λ phages can grow on these cells. LDP were prepared on these cells by infecting them with a phage that was not sensitive to *imm*λ CI[Ts857] repressor-dependent immunity. Accordingly, LDP were prepared by infecting pcIpR-GOI-timm transformed cells with hereroimmune λ*imm*434 phages, e.g., as λ*imm*434*cI* was used to prepare LDP in [26,27], λ*imm*434(18,12)P22 used to prepare LDP-DSE [28] and herein, cells with the pcIpR-GOI-timm expression plasmid were infected with phages λ*imm*434*D*am123 or λ*cI*857*D*am123.

### 2.6. Complementation Methodology 

A total of 594 cells transformed with *D* or *D-*fusion pcIpR-GOI-timm plasmids were induced for GOI expression from about 36–37 °C [29]. The *D* amber phages that were either spotted on, stripped on, or plated on lawns of 594[pcIpR-GOI-timm] cells were complemented at essentially the same plating efficiency when the agar overlay plates were incubated at any temperature between ~36 and 37 °C to 42 °C. The plaques formed on these cells incubated between 37 and 42 °C were identical in size. Full GOI expression in 594[pcIpR-GOI-timm] was observed above 39 °C (Table 1 in [31]). In the present study (e.g., Figure 4) we observed an equivalent efficiency of plating of λ*imm*434*D*am123 on either 594[pcIpR-*D*coe-YML-timm = p674] or 594[pcIpR-*D*coe-timm = p613] cells at culture growth temperatures between 37 and 42 °C. Based on the observations related to gpD-GFP expression [31] and λ*imm*434*D*am123 plating efficiency, we assumed that the gpD and gpD-fusions did not need to be expressed at a high level to effectively complement the *D* amber mutation carried by the infecting *imm*434 phage. Spotted, stripped or plated λ*imm*434*D*am123 does not form plaques on 594[pcIpR-*D*-timm] or 594[pcIpR-*D*coe-YML-timm] cells when they are incubated at 35 °C (or at lower temperatures) even though the *imm*434 phage remains insensitive to *imm*λ repression. 

### 2.7. Complementation Versus Reversion, Versus Marker rescue

#### 2.7.1. D-Amber Reversion

When high concentrations of λ*imm*434*D*am123 were spotted or stripped on nonpermissive host 594 cells in agar overlay and incubated, a few large plaques were observed in the regions of highest spotted phage concentration (see Figure 4, 0.5-dilution spots on 594). Since the cells contain no *D* gene, these pfu arise from spontaneous reversion, measured at a frequency of 3 × 10^−7^ to 1 × 10^−6^ per input phage in the lysate. 

#### 2.7.2. D-Amber Marker Rescue

In addition to simple complementation by the plasmid-expressed *D*-fusion (for the *D*-defect of the infecting phage), the infection of cells with a plasmid encoding a *D*-fusion gene introduces the possibility for phage-plasmid marker rescue. In this situation, the *D*^+^ portion of the *D*-fusion gene can be acquired by recombinational transfer of the gene segment from the plasmid into the phage genome. The frequency for appearance of these newly generated *D*^+^ phage recombinants is determined by examining the reversion frequency for a phage burst released from the infected 594[pcIpR-*D*-fusion-timm] cells. The appearance of pfu on the non-permissive 594 host cells can only arise by either a reversion mutation within the infecting phage genome, or via marker rescue. This is a rare event compared to the much higher frequency for simple complementation. No *D*^+^ pfu were observed for the quadruplicate spots (only one lane is shown) for the 0.5 dilution lysate spotted on 594[p613] or 594[p674] cell lawns at 30 °C. Marker rescue was observed upon repeated serial infections to make high-titer LDP-SEV (i.e., making a primary lysate of λ*imm*434*D*am123 on 594[p674] cells at 39 °C, and then using the resulting phage lysate to prepare a secondary lysate on same host). When assayed, these LDP-SEV preps showed the presence of *D*^+^ recombinants in various lysate preparations at frequencies between 0.002 and 0.0045. Thus, even if these LDP-SEV preparations were >99.5% *D*am123, the fraction that became *D*^+^ arose at >1000× the reversion frequency for λ*imm*434*D*am123 grown up on TC600, which is likely due to marker rescue carry-through during *repeated* serial infections at 39 °C. This suggests two important criteria for making large preps of SEV: (a) do not multiply (serially)-infect transformed cells with a *D*-amber phage (i.e., use only the single infection strategy described in Supplement), and (b) engineer for use, infecting *imm*434 phages that are precisely deleted for *D* so that the possibility for marker rescue is eliminated. 

#### 2.7.3. Complementation Temperature

We previously reported (Tables 4, 5 and Figure 2 in [29]), that because of incomplete repression of *pR* transcription from pcIpR-GOI-timm plasmids, complementation by the GOI can be measured at lower temperatures than 39 °C. Using *imm*434, rather than *imm*λ, phages to detect complementation infection from pcIpR-GOI-timm plasmids bypasses the necessity for *imm*λ phage to completely escape *imm*λ repression by CI[Ts]. Complementation via weak expression of the GOI has been observed as low as 30 °C (data for the highly toxic lambda P protein, Table 4 in [29]) when only trace levels of the GOI are required; at a far lower temperature than where *imm*λ repression is lost. In this situation, the 594[pcIpR-*P*-timm] cells were grown up at 25 °C (not 30 °C) to avoid cell toxicity from weak *P* expression. Thus, full complementation is dependent on the level of the GOI expressed at a given temperature that provides *enough* protein activity to sustain a phenotype, e.g., at 37 °C 594[pcIpR-*D*-YML-timm] cells produce enough *D-*fusion protein to yield infectious LDP, but not enough is made at 35 °C.

### 2.8. Protein Gels and Western Blots

All phage preparations were banded to equilibrium in an isopycnic CsCl gradient (starting average density 1.5 g/cc) to remove any contaminating or associated proteins (excluded from the gradient) and the separated phage band was collected and dialyzed (5000:1) against phosphate buffered saline (PBS: 0.0036 M KCl, 0.0014 M KH_2_PO_4_, 0.136 M NaCl, 0.004 M Na_2 hr_PO_4_, pH 7.4) to remove CsCl. The dialyzed phage samples (5 or 10 µL) were mixed with Novex Tricine -SDS sample buffer (2×) and NuPAGE Reducing Agent (10×) (Invitrogen) and heated as recommended at 85 °C for 130 s and immediately loaded onto Novex precast denaturing 10–20% Tricine slab gels (Invitrogen), 1× Novex Tricine SDS running buffer, with electrophoresis for about 1 h at 130 V. Gels were stained with Oriole fluorescent gel stain (Bio-Rad) and copied in a Bio-Rad Chem Doc using UV light excitation specific for Oriole stain. A 0.2 micron PVDF membrane was treated for 1 min with methanol, then for 15 min with transfer buffer (100 mL of 10X buffer [144 g glycine, 30.2 g Tris base, 900 mL high purity H_2_O], 200 mL methanol, 700 mL H_2_O). The parallel gel was electrophoretically blotted overnight to the membrane at 4 °C (using a Bio-Rad transfer cell) at 10–30 mA constant current (gel to anode, blot to cathode). The blot was removed and blocked by incubating at ambient temperature in 5 mL TBST (TBS with addition of 1 mL Tween 20/L) with 2% bovine serum albumin (BSA) for 1 h. The blocking solution was removed and the blotted membrane was incubated overnight at 4 °C with primary anti-His-gpD mouse antibody (1:1000) in TBST including 3% BSA. The blot was rinsed 3× with TBST and incubated with secondary rabbit anti-mouse IgG (1:1000) conjugated with horseradish peroxidase (HRP) for 1 h in TBST including 5% skim milk powder, rinsed 3× with TBST, and then incubated about 1 min and developed with Bio-Rad enhanced chemiluminescence (ECL, luminol and H_2_O_2_) and the gel bands reacting with His-gpD mouse antibody recorded using Bio-Rad Chem Doc.

## 3. Results and Discussion

### 3.1. Temperature Dependent Gene Expression from Plasmid pcIpR-GOI-Timm

**Figure 3:**
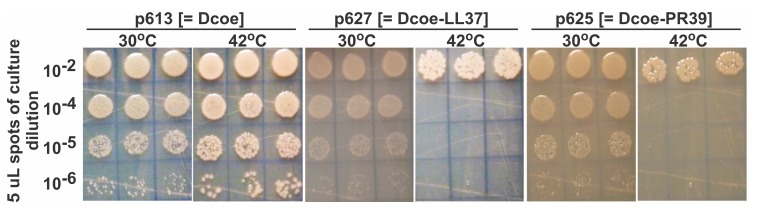
The expression of the *D*-fused pig PR39 [36], or human LL37 [32,33,34,35] cathelicidins killed/prevented the growth of *E. coli* cells expressing the *D*-fusions. Single colonies of cells with plasmid p613 (pcIpR-*D*coe-timm), p625 (pcIpR-*D*coe-PR39-timm, where *D* was fused to a 42 AA pig cathelicidin), and p627 (pcIpR-Dcoe-LL37-timm, where gpD was fused to the 39 AA human cathelicidin) were inoculated into LB-Amp50 medium (Methods and grown at 30 °C in shaking water bath to stationary phase. A 1-mL aliquot was removed and serially diluted into buffer (Methods), and 5-uL aliquots of the dilutions were spotted in quintuplicate onto LB-Amp50 agar plates, one heated to 30 °C and the other to 42 °C, and then moved to the respective incubators for the spots to dry with lids ajar, then inverted with continued incubation overnight. Titers were determined by averaging the cfu (visualized by dissecting microscope) per five spots X dilution factor X 200.

Transcription initiation from *pR* promoter of plasmid pcIrR-GOI-timm, Figure 2, is negatively regulated by the encoded, conditionally active, lambda temperature sensitive CI[Ts857] repressor. In cells grown at 25 °C (but usually at or below 30 °C) there is essentially no transcription initiation from *pR.* Trace transcription (downstream GOI expression) can occur between 30 and 35 °C, but this is only detected if a very highly toxic gene product, or one that can complement at very low abundance, is cloned as the GOI [29]. Under most conditions, the expression of the GOI is not detected by complementation between 30 to 35 °C. In cells incubated at about 36 to 37 °C, complementation activity by the GOI becomes apparent, and the expression of the GOI increases as the culture temperature is increased to 42 °C. For example, when *E. coli* cells with plasmid pcIpR-*D*-GFP-timm were grown at 30 °C, then upshifted for 120 min to 37, 39, or 42 °C, respectively, 7.3, 30, and 721 fluorescence units were observed [31], i.e., a ~100-fold increase in gpD-GFP expression occurred between 37 and 42 °C. [The fluorescence of colony forming units (cfu) arising at 30, 37, 39 and 42 °C is shown in Figure 3 of reference [26].]

### 3.2. Evaluating D-Fusion Cathelicidins for Cellular Toxicity

The toxicity of short *D*-fusions to human (LL37) and porcine (PR39) cathelicidins, expressed from plasmid constructs described in Table 1, was evaluated in non-infected *E. coli* cells, Figure 3. These AMPs were chosen because they contain no cysteines. Their antibacterial activity, in comparison to defensin polypeptides, does not depend on the oxidative formation of three specific intra-peptide sulfhydryl bonds within the cytoplasm of *E. coli*, suggested to be a strongly reducing environment [41]. The expression of gpD, gpD-LL37 and gpD-RP39 *D*-fusions individually cloned into plasmid pcIpR-GOI-timm and transformed into *E. coli* cells is temperature-dependent. The transformed cells were grown up at 30 °C and then spotted to agar plates incubated at either 30 or 42 °C. The expression of gpDcoe was nontoxigenic in cells grown at 42 °C (compare two left panels, Figure 3). On the other hand, both gpDcoe-PR39 and gpDcoe-LL37 expression proved highly toxic for cells incubated at 42 °C. This demonstrates that the cathelicidins each formed a biologically active conformation when fused to gpD.

We assumed that these AMPs would cause lysis of the *E. coli* host cells. Table 2 shows that cell killing was not caused by the D-fused cathelicidin AMP inhibiting protein synthesis, or causing cell lysis, since the culture absorbance A_575 nm_ increased during 140 min incubation at 42 °C (Figure S2). This suggests that neither protein nor cell wall synthesis were inhibited by the expression of the D-fused cathelicidin. This situation is comparable to cells blocked for replication, but which continue to show an increase in culture absorbance due to their formation of filaments. Further studies are needed to explain why cells that internally express a cathelicidin-fused gpD carrier protein do not form colonies. In a related study, cell extracts (obtained at 150 min) from the D-cathelicidin induced cells had no lytic or growth-inhibitory activity when applied to a spread lawn of growing *E. coli* cells. Apparently, their antimicrobial activity depends on gaining-entry into a cell, which may be prevented by fusion to gpD. Studies with the α-defensin HNP1 [42] showed that at bactericidal concentrations, it could permeabilize the outer and inner membrane of *E. coli* and causatively impede DNA, RNA, and protein synthesis.

### 3.3. Temperature-Dependent Complementation for Phage Growth in D-Defective Phage mutant by Plasmids Expressing gpD or gpD-Fusions

**Figure 4:**
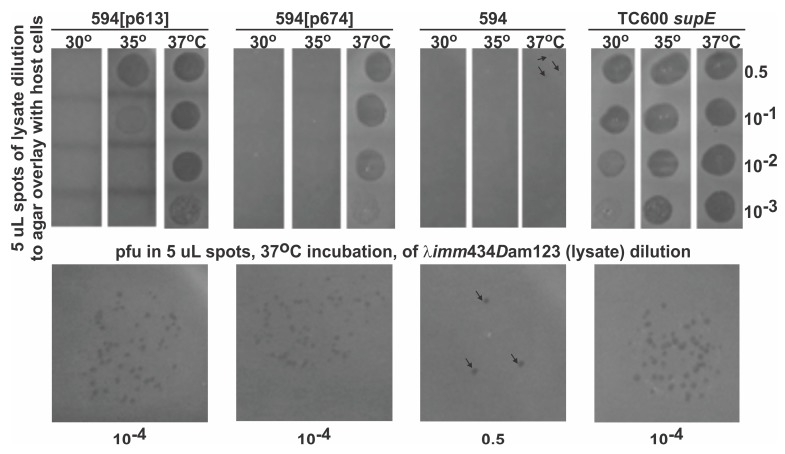
Plating of λ*imm*434*D*am123 spotted on overlaid host cells incubated at 30, 35 and 37 °C. *Top panel:* Representative ability of four host cells to support phage plating (indicated by lysis areas) at three incubation temperatures. (Only one of the side-by-side quintuplicate results is shown per host and incubation temperature.) The dilutions of the phage lysate from which 5 ul aliquots were taken are indicated on the right side. The label on the left side describes the spotted lysate dilutions. The permissive strain TC600 *supE* encodes a modified tRNA that permits some translational read-through of the *D*am123 mutation carried by the agar-spotted infecting phage λ*imm*434*D*am123. The lysate titer of the spotted phage was ~2 × 10^8^ pfu/mL, prepared on TC600 host cells (refer to Section 2.6). Cells for the nonpermissive host, strain 594, were *unable* to suppress the growth defect of the spotted λ*imm*434*D*am123 phage lysate. *Bottom panel:* Individual plaques arising within a 5ul spot per indicated lysate dilution (shown below panels). Phage acquiring a reversion of the *D*am123 mutation, converting it to *D*^+^ appear within the lysate at frequency of ~1 × 10^−6^ or less (Section 2.7.1). Three revertants appear as plaque forming units (pfu) on 594 cells (see arrows to 3 pfu on the 0.5 dilution of spotted phage; no revertant plaques were seen within the other four parallel spots – not shown). The plasmid p674 represents a simplified version of an earlier plasmid used to prepare a string of beads LDP-DSE that presented three immunogenic disease specific epitopes from PrP, i.e., AAs 130-140 [YML]; 163-170 [RL]; and 169-178 [YRR] fused, in single copy on one 116 amino acid fusion to lambda gene *D* (Figure 1 in [28]).

The effective temperature that the expression plasmid can complement a D-deficient phage for plaque formation (i.e., one with amber mutation in *D*) was examined, Figure 4. The spotted phage lysate was able to infect and reproduce at full plating efficiency on TC600 cells grown at 35 and 37 °C (right panel, Figure 4, showing somewhat reduced plating efficiency when the cells were grown at 30 °C), but not on the nonpermissive 594 cells. 

When the *D* amber phage is plated on 594 cells *transformed* with a plasmid expressing either *D* [p613] or a *D*-fusion gene [p674] it becomes possible to measure if the plasmid-expressed gpD or the gpD-fusion can fully complement for the spotted infecting phages defect in *D* and permit sufficient capsid expansion to generate viable phage particles. The plasmid expressed gpD or gpD-fusion needs to produce enough of the complementing protein to enable expansion of the capsid for each infectious phage particle formed, i.e., optimally ~420 monomers of gpD to form ~140 D-trimers. The 594[p613] cells expressing codon optimized gpD (= gpDcoe) and the 594[p674] cells expressing the gpD-fusion protein (Dcoe-YML) fully suppressed the defect in phage λ*imm*434*D*am123 when incubated at 37 °C. The same cells did not express gpD or the gpD-fusion when incubated at 30 °C. The cutoff for efficient *D* expression and complementation by the plasmid expression vector is about 37 °C, below which insufficient *D* expression arises to permit the formation of viable phage particles. 

### 3.4. Evaluating the Influence of D-fusion Size and Toxicity on LDP Production

Short *D*-fusions to cathelicidins or to other AMPs including human α- and β-defensins were evaluated for their influence on cell viability and ability to complement for LDP formation, Table 3. Six Dcoe-fusion constructs, where the gene fusion was to the COOH terminal end of gpD, were highly toxic when the cells were shifted from 30 to 42 °C. This includes fusions to the α-defensin HD5, two β-defensins HBD3 and DEFB126-Δ (a synthetic version of the sperm-coating defensin DEFB126 deleted for its glycosylation sites) and the two cathelicidins LL37 and PR39. The His-Dcoe and the 17-AA fusion that included the 11 amino acid DSE epitope termed YML reduced cell viability by 11–20-fold.

The high toxicity of the gpD-fused α- and β- defensins (gpD-HD5, gpD-HBD3 and gpD- DEFB126-Δ) expressed in cells never infected with lambda phage can be explained by one of two possibilities: (i) either these COOH-fused defensin peptides each undergo the correct formation of three intramolecular disulfide bonds (Figure S3) after their translation within the cytoplasm of *E. coli* cells [41] that were not impaired for synthesis of either thioredoxin or glutathione (i.e., the cells remained competent in *trxB* and *gor* genes); or (ii) that the reduced versions without disulfide bonds are highly toxigenic. There is evidence for the β-defensin HBD1 that *only* after the reduction of its disulfide-bridges does it become a potent AMP against opportunistic pathogens [43]. The possibility that correct disulfide bond formation can occur during the cytosolic assembly of lambda display particles in *E. coli* is supported by the studies of Gupta et al. [17]. They argued that the conditions resulting in cell lysis accompanying lambda phage production could be responsible for providing a cellular oxidizing environment, but this cannot explain our results with uninfected cells. When the gpD-fusions (i.e., both to the cathelicidins lacking disulfide bonds and to the defensins) were made to the NH_2_-terminal end of gpD, the *D*-fusion-expression-toxicity was fully suppressed. The only NH_2_-terminal *D*-fusion that was somewhat toxic (~11-fold) was His-Dcoe. gpD fused to the α-defensin HNP1 was not toxic, nor did it complement for the infecting phages defect in *D*. In reviewing the synthetic sequences for all the prepared D-fusions clones, it was noted that D-HNP1 was prepared without its NH_2_-terminal “A” so that the 6 AA spacer at the COOH-terminal end of gpD was directly joined to the HNP1 CYCIP… coding sequence (plasmid p615, Table 1 and Table 3). The same applied to the NH_2_-terminal HNP1 addition to gpD (plasmid p626). We speculate that (i) either the deleted “A” may be essential for the biological activity of α-defensin HNP1—perhaps for correct disulfide bond formation (compare HNP1 to α-defensin HD5 which encodes four AAs (ARAT) ahead of the first “C”, Figure S3), or (ii) an oxidized version of the HNP1-to-gpD fusions was formed and persisted in the expression cells, which did not undergo subsequent reduction of the disulfide-bridges, which is necessary [43] to form an active antimicrobial.

The greater cellular toxicity of gpDcoe-YML to that of gpD-YML, i.e., 20 to 1 [comparing plasmids p675 encoding D(wild type)-YML and p674 encoding Dcoe-YML: Table 3 column for Relative Culture Viability at 42 °C], may reflect a higher level of the gpDcoe-YML fusion *expression* in cells. The Dcoe (codon optimized) sequence was synthetically altered for 55 of the 110 codons in *D* [26,27] to improve translation. We suggest a hypothesis that wild type *D* encoded within the lambda genome may have evolved to ensure a reduced level of *D* expression. This could explain why cells with pcIpR-Dcoe-timm or pcIpR-Dcoe-YML-timm that are marginally induced for *D* expression at 37 °C can efficiently complement the growth of λ*imm*434*D*am123 phage (compare to D-GFP expression, Section 3.1).

The ability of the D-fusions to complement for growth of λ*imm*434*D*am123 and substitute for the defective *D* encoded on the phage genome is shown in Table 3 (right column). Greatly reduced phage plating was observed when the expressed *D*-fusion protein was highly toxic to cells. A high level of suppression of the *D* amber mutation was seen in cells with plasmids expressing Dcoe or the COOH-fusions Dcoe-YML and D(wild type)-YML. Very poor suppression occurred for the NH_2_-terminal fusion 15AA-Dcoe. No suppression was afforded by NH_2_-terminal His-Dcoe fusion. Except for D-HNP1, both the toxicity and suppression data suggest that polypeptide additions to the COOH-terminal end of gpD can establish antimicrobial activity, whereas the additions to the NH_2_-terminal end of gpD were somehow handicapped. This suggests that polypeptides fused to the COOH-terminal end of gpD have a higher likelihood of achieving a biologically relevant conformation.

**Figure 5:**
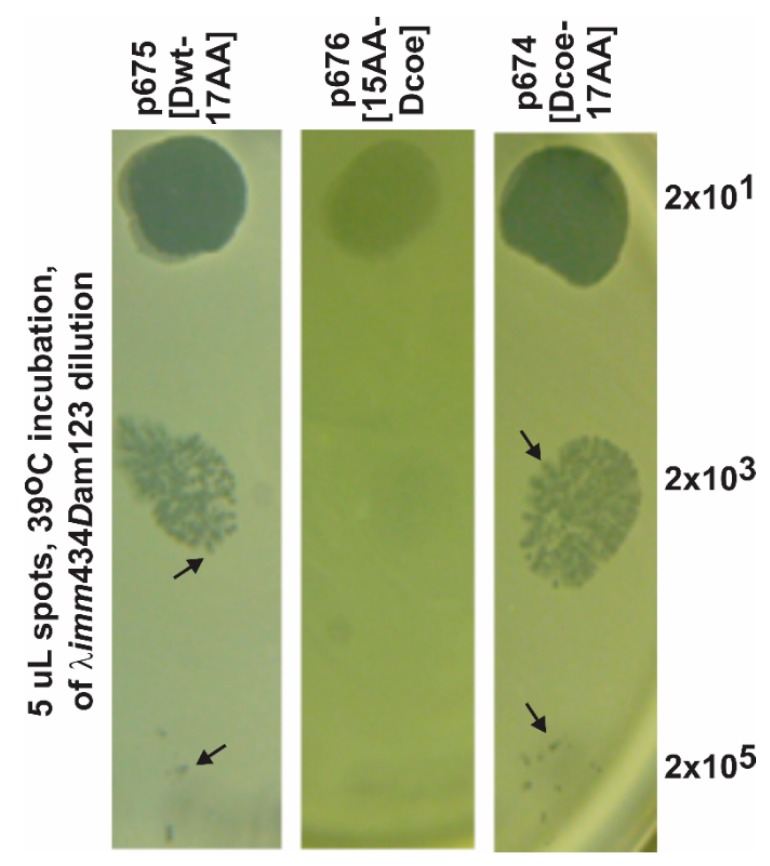
Complementation for phage growth by plasmid expression vectors expressing three distinct *D*-fusions. (Refer to Table S1 for quantitative results). Each *D*-fusion construct included an 11 amino acid (GYMLGSAMSRP, termed YML) single epitope from the sequence of the cervid prion PrP protein [28].

The ability of three plasmid version encoding the 11 amino acid DSE string YML to complement infecting phage with a *D* amber mutation is shown (Figure 5). The YML-epitope was joined either to the NH_2_-terminal end of gpD or joined to a 6 amino acid linker at the COOH-terminal end of gpD (Table 1 and Table 3). The NH_2_-terminal fusion complemented poorly for gpD (indeed, maybe only cell killing was observed), whereas the COOH-terminal fusions with *D* or *D*coe permitted individual plaque formation at an efficiency of ~90% or better (compared to plating efficiency on TC600 *supE*), Table S1.

**Figure 6:**
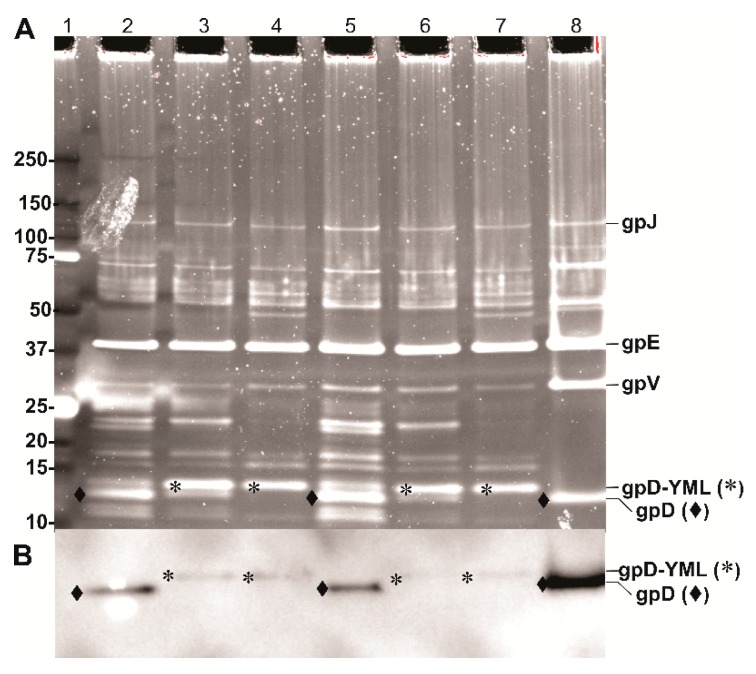
Display of gpD-YML epitope by λ*cI*857*D*am123 and λ*imm*434*D*am123 phages. Parallel, side by side protein electrophoresis runs of dialyzed CsCl-banded phage preps. (**A**) Oriole fluorescent gel stain (Bio-Rad) with UV light excitation and (**B)** Western blot using primary anti-His-gpD mouse antibody (kindly provided by Dr. Philip Griebel, VIDO/Intervac, University of Saskatchewan) with chemiluminescence detection of the secondary rabbit anti-mouse IgG (HRP conjugated). Lanes for **A** and **B**: Lane 1: 1 µL of Bio-Rad Precision Plus Protein Standards (10 Kd to 250 kDa). Lanes 2 and 5: λ*cI*857*D*am123 infecting *E. coli* strain 594[pcIpR-*D*coe-timm] = p613 induced for *D*coe expression from plasmid p613 at 38 °C. Lanes 3 and 6: λ*cI*857*D*am123 infecting *E. coli* strain 594[pcIpR-*D*coe-YML-timm] = p674, induced for *D*coe-YML expression from plasmid p674 at 38 °C. Lanes 4 and 7: λ*imm*434*D*am123 infecting *E. coli* strain 594[pcIpR-*D*coe-YML-timm] induced for *D*coe-YML expression from plasmid p674 at 38 °C. Lane 8, λ*imm*434*cI*#5 (wild type for *D*) infecting TC600[pcIpR-*D*coe-BVDV-ver2-timm] = p521, induced for *D*coeVer2 expression from plasmid p521 at 42 °C.

Phage lysates were prepared by complementation, as illustrated by the complementation data shown for p674 [Dcoe-17 AA] = gpD-YML (Figure 5). The lysates were prepared by the “single-burst LDP-vaccine and SEV production” methodology (described in Supplement). The phage particles present in these lysates were banded in CsCl, and the recovered band was dialyzed, disrupted by heat, and the capsid protein bands were separated on polyacrylamide gels (Figure 6). The distinction between gpD and the 17 AA-larger gpD-YML phage capsid protein is shown by differences in gel migration, Figure 6A. The banded phage from the infections shown for lanes 3,4,6,7 can only employ the gpD-YML fusion protein for addition to the capsid. The results are confirmed in the Western blot of the same gel, Figure 6B, using primary mouse antibody prepared to purified His-gpD [28]. The display of the YML epitope from gpD reduced the affinity (fainter bands) of anti-His-gpD for gpD-YML, compared to gpD. 

Since gpD-YML migrates more slowly than gpD, it is possible to see the minor gp*FII* capsid protein band in lanes 3 and 6 when gpD is substituted by gpD-YML. gpFII is not seen when gpD (non-fusion) is present since it is approximately the same molecular weight as gpD, and gpD occupies far more sites on the capsid. The gpFII protein has been suggested to be detected in incomplete full capsid heads [3], and is more prominent in the λ*cI*857*D*am123 infections (i.e., in lanes 3, 6 as compared to lanes 4, 7). The major lambda capsid bands, gpJ, gpE, gpV and gpD are tagged. Other capsid head, tail, and tail fiber bands [44] are visible, likely because the Oriole stain as suggested by the manufacturer is even more sensitive than silver staining. (The additional bands were not visible when Coomassie R protein stain was employed). The distinctions between the λ*cI*857 and λ*imm*434 banding patterns revealed by the Oriole stain require further study to elucidate band distinctions. We sequenced both the capsid *D* and *E* genes for both phages and they were identical, as was the sequence for the *D*am123 mutation.

A future post-antibiotic era in which the acquisition of multiple levels of antibiotic resistance by pathogenic microorganisms will lessen the utility of antibiotics for treating infections can be imagined. Many alternative therapeutic strategies are being explored, including the use of broad-spectrum AMPs and narrow-spectrum phage therapy, where targeted pathogens are infected and lysed by bacterial viruses that have been isolated, characterized, and stockpiled for their unique infection specificity. Ideas to combine these latter two strategies are gaining interest. One approach is to clone a genetic expression system for a broad-spectrum AMP, i.e., promoter, AMP coding unit, transcriptional terminator, within a bacterial virus genome. The idea is that the bacterial virus encoding the AMP (or any bactericidal agent) will infect sensitive bacterial cells within the infected organism, and the lysed recipient cell will release the broad-spectrum AMP, which in turn will indiscriminately kill multiple pathogenic bacterial species within the milieu [45]. This idea is based on the expense involved in synthesizing therapeutic doses of AMP, toxicity considerations, and their relative instability, particularly in serum [46,47]. In this study, we explored an alternative idea either to phage therapy or a phage-expressed bactericidal agent. This was to generate antimicrobials by coating bacterial virus particle capsids with broad spectrum AMP. Phage particles that must survive diverse environmental conditions to remain viable should be much more stable than individual peptides. We are not aware of studies that have examined the conformational stability of AMP linked to phage capsid proteins. We found that the ability of fused polypeptides to form a conformation that can exhibit biological activity cannot be taken for granted. We also came to understand that the fused AMP polypeptides which could attain a biologically active conformation were highly toxic when synthesized within *E. coli*, yet appeared to possess no external lytic activity towards growing *E. coli* cells. It is interesting to speculate whether bacterial mutants can be obtained that are resistant to cytosolic AMP-fusion toxicity. Such mutants may indeed provide a host needed for the preparation of LDP-AMP display particles, as well as providing insight into how AMPs exert bactericidal activity when expressed within a cell.

We previously examined the utility of obtaining high display density LDP for the purpose of producing vaccines to DSE. We noted that the targeted delivery of purified LDP-DSE to intestinal segments of calves induced IgA responses to each of three fused peptide epitopes. The peptides were arranged and displayed as one COOH-terminal 116 amino acid fusion to gpD in a string-of-beads arrangement. These LDP served as a vaccine without the addition of a mucosal adjuvant and confirmed Peyer’s patches as the site for LDP-DSE uptake [28]. The display density of the DSE on these LDP proved to be low. Complementation studies were employed, herein, to explore various length fusions to gpD which would allow for the formation of infectious LDP, i.e., with “sufficient” addition of the gpD-fusion to the capsid to form a viable phage particle in the absence of wild type gpD. This study suggests that the display of short single epitopes, even if somewhat toxic to cells when produced, would result in infectious high-density display particles. Accordingly, we suggest (see Supplementary File S1) abandoning the string-of-beads display approach, with new focus on the utility of generating single-epitope vaccines, e.g., one for each separate bead on the string, all of which can be produced and displayed as a mixture resulting from parallel LDP-DSE infections. 

## 4. Further Considerations and Summary

We examined several aspects of making and expressing NH_2_- and COOH-terminal gpD fusions of DSE, or of polypeptides with antimicrobial activity. We evaluated fusion size, cellular ability to express highly hydrophobic and positively charged gene fusions to gpD, the potential of the fused polypeptide to form an active antimicrobial conformation, the toxicity of the gpD-fusion to the cell, the potential ability of some of the fusion polypeptides to serve as substrates for the formation of three correct intramolecular disulfide bonds, and the potential of the gpD-fusions to complement for the formation of viable phage display particles in the absence of active gpD. 

We joined 11, 15, 36, 41,46, 49, 53 and 85 AA at the NH_2_-end of gpD and 17, 35, 40, 45, 48, 50, and 84 AA to the COOH- end of gpD, plus two fusions were made with AAs fused to both the NH_2_- and COOH-ends of gpD (50 AA to NH_2_- and 12 AA to COOH, and 11 AA to NH_2_- and 55 AA to COOH). With one exception (His-gpD), none of the gpD-fusions with NH_2_-terminal additions were toxic when expressed in *E. coli* cells. The parallel versions with COOH-terminal fusions proved highly toxic, suggesting that these gpD-fused polypeptides can form a conformation that is biologically active. Three constructs where the fused peptides have the potential to form intramolecular disulfide bonds [i.e., gpD-6AA(linker)-fusions to the α-defensin peptide HD5, the β-defensin HBD3 and a synthetic truncated version of the β-defensin DEFB126], attained a biologically active (high-toxicity) conformation. Very possibly, correct triple disulfide bond formation (Figure S3) occurred within the cytoplasm of *E. coli* cells in these three fused defensin polypeptides. We conclude that gene fusions to the COOH-terminal, and not to the NH_2_-terminal end of gpD, are more likely to enable the attainment (or approximation) of the native conformation of a displayed polypeptide. Our results strongly show that cathelicidins and α- and β-defensin antimicrobial polypeptides could retain their antimicrobial activity when fused to gpD. The antimicrobial activities observed were exclusively internal to the cells where they were expressed. We are not aware of other studies fusing antimicrobial polypeptides to a bacterial virus capsid protein.

The predominant model for diverse AMP activity involves their external contact and disruption of the outer cell wall. We suggest that their bactericidal activity could (also) depend upon/involve entry into the cytoplasm. More research into their mode of action is mandated. We were able to generate highly toxic gpD-fusion proteins, but we were misled in thinking that they would exhibit toxicity only when externally applied to a bacterial cell and would not exhibit cytosolic toxicity. The high internal cellular toxicity of some of the gpD-fusion proteins prevented them from complementing infecting phages with defective gpD and, indeed, cell growth was so incapacitated that phage morphogenesis/plating was impossible to observe. We conclude that the antimicrobial activity of the toxic gpD-fusion proteins clearly obstructed one or more essential internal cellular metabolic activities, required both by the cell, and for phage morphogenesis. 

The discovery of highly toxic gpD-antimicrobial fusion proteins provides a challenge for extending their utility, i.e., of getting them, or an expression system for them, *into* diverse pathogenic bacterial cells. It is likely that routine transformation technology would be too inefficient. However, the inclusion of a gpD-fusion-antimicrobial gene, particularly an expression system for gp-D-cathelicidin fusions, within the genome of a therapy phage that uniquely targets a given pathogen seems a strong possibility. DNA injection of a phage genome (with gpD-fusion-insertion) into a bacterial pathogen can serve two goals: upon the expression of the gpD-fusion element from the injected phage genome that gains entry into the target cell (i) the infected target cell will be arrested for growth, and (ii) further phage morphogenesis (and phage-dependent cell lysis) will be prevented.

**Figure 7:**
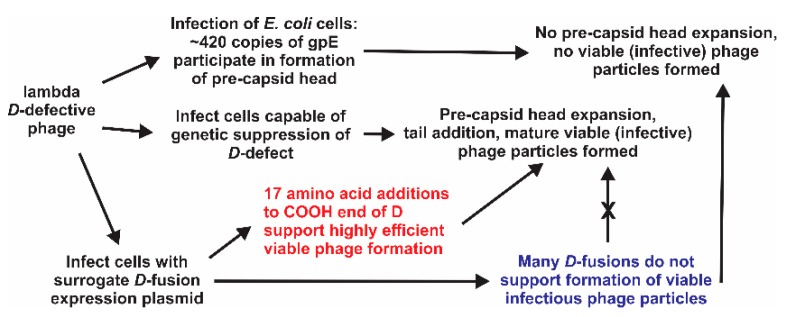
Summary gpD complementation. Fusing short epitopes at the COOH-end of gpD can permit very high efficiency LDP recovery. Displaying single epitopes of about 17 amino acids can provide an efficient method for preparing SEV, avoiding recovery problems that could be encountered when attempting to display larger polypeptide fusions.

We observed the efficient formation of LDP-DSE displaying a short DSE epitope joined to a linker at the COOH end of *D* or *D*coe, even if toxic, summarized in Figure 7. This result suggests that short epitopes fused to gpD and incorporated into LDP-DSE can serve as high display density single epitope vaccines (SEV). We propose that these LDP-DSE particles will be substantially easier to prepare than string-of-beads LDP vaccines, with the advantages of having full density gpD-fusion display and conformationally active single epitope antigenic activity. 

In a companion Supplementary File S1, we provided a hypothesis for banking gene expression cell systems to prepare multiple SEV phage display vaccines on demand and explained how SEV-DSE vaccines can be economically delivered without a need for any refrigeration in the delivery chain. The short-term value of this scheme may be to represent a simple procedure in combination with mouse bioassays for evaluating the immunogenic/protective activity of DSE derived from pathogen proteins.

## Figures and Tables

**Table 1:** Overview of *D*-fusion gene constructs cloned into the pcIpR-GOI-timm expression vector.

*D*-Fusion Name (Plasmid Number)	
COOH-terminal additions: ^a^	NH_2_-terminal additions: ^a^
His-TAGZ-Dcoe-TEV-LL37 (p619) ^b^	LL37-TEV-Dcoe-His (p620)
Dcoe-LL37 (p627) ^c^	LL37-Dcoe (p617) ^c^
Dcoe-PR39 (p625) ^c^	PR39-Dcoe (p623) ^c^
Dcoe-DEFB126ΔC (p618) ^d^	DEFB126ΔC-Dcoe (p622) ^d^
Dcoe-HBD3 (p616)	HBD3-Dcoe (p624)
Dcoe-HD5 (p628)	HD5-Dcoe (p621)
Dcoe-HNP1 (p615) [see text]	HNP1-Dcoe (p626) [see text]
Dcoe-YML (p674)	YML-Dcoe (p676)
D-YML (p675)	His-Dcoe (p614)
Dcoe-5EV2 (p521,p629) ^e^	His-Dcoe^-^ (p614*) ^f^

^a^ COOH-terminal additions: the MTSK…SIV sequence for gpD and gpDcoe (see text) was followed by six amino acid spacer (see text) and then a COOH terminal addition (with the AAs arranged in their standard NH_2_ to COOH orientation). NH_2_-terminal additions: all constructs were of the arrangement M-spacer-TSK…SIV, except for p614, p614* and p676 where no spacer was included. ^b^ TEV (tobacco etch virus) represents the protease recognition sequence ENLYFQX, and the sequence ahead of gene *D* is recognized by the TAGZyme protease (Qiagen) for cleaving the His tag. ^c^ LL37 represents 39 AAs at the COOH-end of hCAP18 gene (167 codons) where the NH_2_-terminal CATHELIN domain is removed. *D*coe-LL37 fuses the sole human cathelicidin, LL37, to the COOH end of the *D*-spacer. *D*coe-PR39 fuses the 42 AA pig PR39 cathelicidin to a 6 AA spacer at the COOH-end of gpD. ^d^ The wild type human sperm defensin DEFB126 is expressed in the epididymis [39,40] and is 111 AAs. The molecule’s 18 COOH-terminal glycosylation sites were deleted (indicated by ΔC) in constructing *D*coe-DEFB126ΔC. The hydrophobic N-terminal end of DEFB126 is highly positively charged. ^e^ Epitopes from BVDV2 E2 protein. ^f^ As for p614, except with C to A transversion at bp 143, P48Q, in Dcoe.

**Table 2:** Culture absorbance change after induction of plasmid-expressed *D*-fusion genes. ^a^

Plasmid In *E. coli*, Encoded *D* or *D*-Fusion Protein	Non-Induces (0 Time)	Non-Induced, 140 min Cell Growth at 30 °C	Induced, 140 min Cell Growth after Shifting Culture to 42 °C
p613, gpDcoe	0.10	0.44	0.79
p627, gpD-PR39	0.14	0.51	0.70
p625, gpD-LL37	0.15	0.71	0.70

^a^ Single colony isolates of strains p613, p625 and p627 (refer to Table 1 and Table 3 and Section 2.3), respectively 594[pcIpR-Dcoe-timm], 594[pcIpR-Dcoe-PR39-timm] and 594[pcIpR-Dcoe-LL37-timm], were inoculated into LB-Amp50 medium and grown up in a 30 °C shaking water bath until reaching an A_575_ of about 0.1. Each culture was split: One-half was returned to the 30 °C shaking bath to maintain repression of the plasmid cloned GOI. The parallel culture was shaken for 15 s at 60 °C (this rapidly brings the culture temperature up to ~39 °C) and then shaken in a 42 °C bath to permit full expression of the plasmid encoded GOI. The A_575_ values were recorded at 60, 90, 120 and 140 min (see Figure S2).

**Table 3:** Assessing if Antimicrobial peptides (AMPs) fused to gpD can complement for lambda infectivity and if the expression of the construct is toxic to *E. coli* cells.

Strain 594[pcIpR-*D*-fusion-timm] Plasmid Construct ^a^	Relative Culture Viability at 42 °C (*D*-fusion Toxicity) ^b^	EOP ^c^ of λ*imm*434 *D*am123 at 42 °C on Assay Strain ^d^
p613: *D*coe	0.65	0.56
p613*: *D*coe^-^ (67 bp deletion within *D*) ^e^	0.79	<0.0001
p614: His-*D*coe (11AA N-terminal)	0.09	<0.0001 ^f^
p614*: His- *D*coe^-^:*(Pro48Glu mutation in *D*)	0.87	<0.0001
p615: *D*coe*-*HNP1 (35AA C-term.)	0.83	<0.0001 ^f^
p626: HNP1*- D*coe (36AA N-term.)	1.00	<0.0001 ^f^
p616: *D*coe-HBD3 (50AA C-term.)	<0.0001	<0.0005
p624: HBD3- *D*coe (53AA N-term.)	0.78	<0.0001 ^f^
p627: *D*coe-LL37 (45AA C-term.)	0.0002	^g^
p617: LL37-*D*coe (46AA N-term.)	0.93	<0.0001 ^f^
p618: *D*coe-DEFB126-Δ (84AA C-term.)	0.0001	^g^
p622: DEFB126-ΔC- *D*coe (85AA N-term.)	0.85	<0.0001 ^f^
p619: His-TAGZ-*D*coe-TEV-LL37 (11AA N-, 55AA C-term.)	0.0003	<0.000 1^f^
p620: LL37-TEV- *D*coe-His (50AA N-, 12AA C-term.)	0.87	<0.0001 ^f^
p628: *D*coe-HD5 (40AA C-term.)	0.0034	<0.0001 ^f^
p621: HD5- *D*coe (41AA N-term.)	0.77	<0.0001 ^f^
p625: *D*coe*-*PR39 (48AA C-term.)	<0.0001	^g^
p623: PR39-*D*coe (49AA N-term.)	0.97	<0.0001 ^f^
p674: *D*coe-YML (17AA C-term.)	0.05	≥0.90 ^h^
p675: *D*(wild type)-YML (17AA C-term.)	0.88	0.93
p676: YML-*D*coe (15AA N-term.)	1.00	0.06

^a^ The AMPs include α- and β-defensins and cathelicicins, with construction details indicated in Table 1 and in Methods. The amino acid fusions were made either at NH_2_-, or COOH-terminal end of *D*. ^b^ Efficiency of plating (EOP) of the 594[pcIpR-D-fusion-timm] *E. coli* cells. Cell titers obtained at 42 °C (where the cloned *D*-fusion gene is fully expressed) were divided by cell titers for the same strain incubated in parallel at 30 °C, where the *D*-fusion gene expression is repressed. This assay measures the relative cell viability change upon cellular expression of the *D*-fusion. ^c^ Complementation assay: EOP of a lysate of phage λ*imm*434*D*am123 with a conditional nonsense mutation in *D* plated on assay strain 594[pcIpR-D-fusion-timm] host cells grown at 42 °C compared to the plating of the same phage on TC600 cells. The lysate of λ*imm*434-*D*am123 was diluted 10^−1^, 10^−2^, 10^−3^, and 10^−4^ in buffer and 5 µL aliquots of the dilutions were spotted in triplicate on agar overlay plates made with 3.0 mL LB top agar and 0.3 mL of a stationary culture of cells grown up in LB-Amp50. When the spots dried on the plates at room temperature they were inverted and incubated overnight at 41–42 °C. ^d^ In parallel assays, plating efficiency was measured to determine if the expressed D-fusion proteins negatively complemented for the ability of D to form infectious phage particles: (i) strain TC600 *supE* was transformed with each of the plasmids and the plating efficiency of λ*imm*434*D*am123 was measured, or (ii) the 594 transformants shown were infected with the *D*^+^ phages λ*imm*434*cI* or λ*imm*434(*18*,*12*)P22 (where lambda genes *O-P* were substituted by genes *18*–*12* of phage P22). In both (i) and (ii) none of the D-fusions negatively complemented for growth of the *imm*434 phages on cells induced for expression of the cloned D-fusion gene. ^e^ The *D* sequence in p613* comprises the first 50 AA (codons intact) plus the first base of codon 51, then 87 codons (AAs) of scrambled sequence terminating in TAG stop, so it is 27 AA longer than the encoded 110 AAs of gpD (before the NH_2_-terminal methionine is removed). ^f^ Faint phage lysis spots were seen for the 10^−1^ dilution spots, but no individual plaques were observed. ^g^ There was too much killing of cells in the overlay to determine if the phage could be complemented. ^h^ These cells supported high-level complementation to λ*imm*434 *D*am123 when agar overlay plates were incubated at 37 °C, 39 °C and 41–42 °C (Table S1); approximating the phage titer on TC600 *supE* host cells at 39 °C.

## Supplementary Materials

The following are available online at https://www.mdpi.com/1999-4915/11/9/869/s1, Figure S1: Coating lambda phage head with gpD or gpD-fusion proteins. Figure S2: Comparison of culture growth after gpD, gpD-LL37 and gpD-PR39 expression. Figure S3: Predicted disulfide bonds formed in *D*-fusion defensins. Table S1: Complementation by plasmid expression vectors of an amber mutation within the capsid decoration protein gpD in two lambdoid phages. Method for Single-burst LDP-vaccine and SEV production. Supplementary File S1: Single epitope vaccines (SEV): a hypothesis and approach using phage display particles made from bacterial virus lambda.

## Abbreviations

LDP: bacteriophage lambda display particles. DSE, disease specific epitope. *D* or *D*coe, bacteriophage lambda capsid decoration gene or a (*co*don *e*nhanced) version. GFP, green fluorescent protein. AAs, amino acids. gpD, the gene product of *D*. SEV, single epitope vaccines. AMPs, antimicrobial peptides, where LL37 and PR39 are human and porcine cathelicidins; HNP1 and HD5 are human α-defensins; and HBD3 and DEFB126 are human β-defensins.

## References

[1] Casjens S.R., Hendrix R.W. (1974). Locations and amounts of major structural proteins in bacteriophage lambda. J. Mol. Biol..

[2] Imber R., Tsugita A., Wurtz M., Hohn T. (1980). Outer surface protein of bacteriophage lambda. J. Mol. Biol..

[3] Georgopoulos C., Tilly K., Casjens S., Hendrix R.W., Roberts J.R., Stahl F.S., Weisberg R.A. (1983). Lambdoid phage head assembly. Lambda ii.

[4] Sternberg N., Hoess R.H. (1995). Display of peptides and proteins on the surface of bacteriophage lambda. Proc. Natl. Acad. Sci. USA.

[5] Sternberg N., Weisberg R. (1977). Packaging of coliphage lambda DNA. Ii. The role of the gene d protein. J. Mol. Biol..

[6] Ansuini H., Cicchini C., Nicosia A., Tripodi M., Cortese R., Luzzago A. (2002). Biotin-tagged cdna expression libraries displayed on lambda phage: A new tool for the selection of natural protein ligands. Nucleic Acids Res..

[7] Cicchini C., Ansuini H., Amicone L., Alonzi T., Nicosia A., Cortese R., Tripodi M., Luzzago A. (2002). Searching for DNA-protein interactions by lambda phage display. J. Mol. Biol..

[8] Cortese R., Monaci P., Luzzago A., Santini C., Bartoli F., Cortese I., Fortugno P., Galfre G., Nicosia A., Felici F. (1996). Selection of biologically active peptides by phage display of random peptide libraries. Curr. Opin. Biotechnol..

[9] Kong B., Ma W.J. (2006). Display of aggregation-prone ligand binding domain of human ppar gamma on surface of bacteriophage lambda. Acta Pharmacol. Sin..

[10] Maruyama I.N., Maruyama H.I., Brenner S. (1994). Lambda foo: A lambda phage vector for the expression of foreign proteins. Proc. Natl. Acad. Sci. USA.

[11] Mikawa Y.G., Maruyama I.N., Brenner S. (1996). Surface display of proteins on bacteriophage lambda heads. J. Mol. Biol..

[12] Niwa M., Maruyama H., Fujimoto T., Dohi K., Maruyama I.N. (2000). Affinity selection of cdna libraries by lambda phage surface display. Gene.

[13] Santi E., Capone S., Mennuni C., Lahm A., Tramontano A., Luzzago A., Nicosia A. (2000). Bacteriophage lambda display of complex cdna libraries: A new approach to functional genomics. J. Mol. Biol.

[14] Santini C., Brennan D., Mennuni C., Hoess R.H., Nicosia A., Cortese R., Luzzago A. (1998). Efficient display of an hcv cdna expression library as c-terminal fusion to the capsid protein d of bacteriophage lambda. J. Mol. Biol..

[15] Vilchez S., Jacoby J., Ellar D.J. (2004). Display of biologically functional insecticidal toxin on the surface of lambda phage. Appl. Environ. Microbiol..

[16] Zucconi A., Dente L., Santonico E., Castagnoli L., Cesareni G. (2001). Selection of ligands by panning of domain libraries displayed on phage lambda reveals new potential partners of synaptojanin 1. J. Mol. Biol..

[17] Gupta A., Onda M., Pastan I., Adhya S., Chaudhary V.K. (2003). High-density functional display of proteins on bacteriophage lambda. J. Mol. Biol..

[18] Zanghi C.N., Lankes H.A., Bradel-Tretheway B., Wegman J., Dewhurst S. (2005). A simple method for displaying recalcitrant proteins on the surface of bacteriophage lambda. Nucleic Acids Res..

[19] Zanghi C.N., Sapinoro R., Bradel-Tretheway B., Dewhurst S. (2007). A tractable method for simultaneous modifications to the head and tail of bacteriophage lambda and its application to enhancing phage-mediated gene delivery. Nucleic Acids Res..

[20] Gao J., Wang Y., Liu Z. (2010). Phage display an its application in vaccine design. Ann. Microbiol..

[21] Ren Z.J., Lewis G.K., Wingfield P.T., Locke E.G., Steven A.C., Black L.W. (1996). Phage display of intact domains at high copy number: A system based on soc, the small outer capsid protein of bacteriophage t4. Protein Sci..

[22] Ren Z.J., Tian C.J., Zhu Q.S., Zhao M.Y., Xin A.G., Nie W.X., Ling S.R., Zhu M.W., Wu J.Y., Lan H.Y. (2008). Orally delivered foot-and-mouth disease virus capsid protomer vaccine displayed on t4 bacteriophage surface: 100% protection from potency challenge in mice. Vaccine.

[23] Sathaliyawala T., Rao M., Maclean D.M., Birx D.L., Alving C.R., Rao V.B. (2006). Assembly of human immunodeficiency virus (hiv) antigens on bacteriophage t4: A novel in vitro approach to construct multicomponent hiv vaccines. J. Virol..

[24] Shivachandra S.B., Rao M., Janosi L., Sathaliyawala T., Matyas G.R., Alving C.R., Leppla S.H., Rao V.B. (2006). In vitro binding of anthrax protective antigen on bacteriophage t4 capsid surface through hoc-capsid interactions: A strategy for efficient display of large full-length proteins. Virology.

[25] Wu J., Tu C., Yu X., Zhang M., Zhang N., Zhao M., Nie W., Ren Z. (2007). Bacteriophage t4 nanoparticle capsid surface soc and hoc bipartite display with enhanced classical swine fever virus immunogenicity: A powerful immunological approach. J. Virol. Methods.

[26] Hayes S., Gamage L.N., Hayes C. (2010). Dual expression system for assembling phage lambda display particle (ldp) vaccine to porcine circovirus 2 (pcv2). Vaccine.

[27] Gamage L.N., Ellis J., Hayes S. (2009). Immunogenicity of bacteriophage lambda particles displaying porcine circovirus 2 (pcv2) capsid protein epitopes. Vaccine.

[28] Gonzalez-Cano P., Gamage L.N.A., Marciniuk K., Hayes C., Napper S., Hayes S., Griebel P.J. (2017). Lambda display phage as a mucosal vaccine delivery vehicle for peptide antigens. Vaccine.

[29] Hayes S., Erker C., Horbay M.A., Marciniuk K., Wang W., Hayes C. (2013). Phage lambda p protein: Trans-activation, inhibition phenotypes and their suppression. Viruses.

[30] Hayes S., Rajamanickam K., Hayes C. (2018). Complementation studies of bacteriophage lambda o amber mutants by allelic forms of o expressed from plasmid, and o-p interaction phenotypes. Antibiotics.

[31] Rajamanickam K., Hayes S. (2018). The bacteriophage lambda cii phenotypes for complementation, cellular toxicity and replication inhibition are suppressed in cii-oop constructs expressing the small rna oop. Viruses.

[32] Braff M.H., Hawkins M.A., Di Nardo A., Lopez-Garcia B., Howell M.D., Wong C., Lin K., Streib J.E., Dorschner R., Leung D.Y. (2005). Structure-function relationships among human cathelicidin peptides: Dissociation of antimicrobial properties from host immunostimulatory activities. J. Immunol..

[33] Vandamme D., Landuyt B., Luyten W., Schoofs L. (2012). A comprehensive summary of ll-37, the factotum human cathelicidin peptide. Cell Immunol..

[34] Yang D., Chertov O., Oppenheim J.J. (2001). Participation of mammalian defensins and cathelicidins in anti-microbial immunity: Receptors and activities of human defensins and cathelicidin (ll-37). J. Leukoc. Biol..

[35] Zanetti M. (2004). Cathelicidins, multifunctional peptides of the innate immunity. J. Leukoc. Biol..

[36] Sang Y., Blecha F. (2009). Porcine host defense peptides: Expanding repertoire and functions. Dev. Comp. Immunol..

[37] Selsted M.E., Harwig S.S., Ganz T., Schilling J.W., Lehrer R.I. (1985). Primary structures of three human neutrophil defensins. J. Clin. Investig..

[38] Schneider J.J., Unholzer A., Schaller M., Schafer-Korting M., Korting H.C. (2005). Human defensins. J. Mol. Med..

[39] Tollner T.L., Bevins C.L., Cherr G.N. (2012). Multifunctional glycoprotein defb126—A curious story of defensin-clad spermatozoa. Nat. Rev. Urol..

[40] Tollner T.L., Venners S.A., Hollox E.J., Yudin A.I., Liu X., Tang G., Xing H., Kays R.J., Lau T., Overstreet J.W. (2011). A common mutation in the defensin defb126 causes impaired sperm function and subfertility. Sci. Transl. Med..

[41] Bessette P.H., Aslund F., Beckwith J., Georgiou G. (1999). Efficient folding of proteins with multiple disulfide bonds in the escherichia coli cytoplasm. Proc. Natl. Acad. Sci. USA.

[42] Lehrer R.I., Barton A., Daher K.A., Harwig S.S., Ganz T., Selsted M.E. (1989). Interaction of human defensins with escherichia coli. Mechanism of bactericidal activity. J. Clin. Investig..

[43] Schroeder B.O., Wu Z., Nuding S., Groscurth S., Marcinowski M., Beisner J., Buchner J., Schaller M., Stange E.F., Wehkamp J. (2011). Reduction of disulphide bonds unmasks potent antimicrobial activity of human beta-defensin 1. Nature.

[44] Hendrix R.W., Duda R.L. (1992). Bacteriophage lambda papa: Not the mother of all lambda phages. Science.

[45] Lemon D.J., Kay M.K., Titus J.K., Ford A.A., Chen W., Hamlin N.J., Hwang Y.Y. (2019). Construction of a genetically modified T7 select phage system to express the antimicrobial peptide 1018. J. Microbiol..

[46] Gordon Y.J., Romanowski E.G., McDermott A.M. (2005). A review of antimicrobial peptides and their therapeutic potential as anti-infective drugs. Curr. Eye Res..

[47] Knappe D., Henklein P., Hoffmann R., Hilpert K. (2010). Easy strategy to protect antimicrobial peptides from fast degradation in serum. Antimicrob. Agents Chemother..

