# Supplementary material for: Bacterial Virus Lambda Gpd-Fusions to Cathelicidins, α- and β-Defensins, and Disease-Specific Epitopes Evaluated for Antimicrobial Toxicity and Ability to Support Phage Display
Source: Viruses. 2019 Sep 17;11(9):869. doi: 10.3390/v11090869 (PMC6784203; doi:10.3390/v11090869)
Supplement: Supplementary file 1 [file viruses-11-00869-s001.pdf]

Supplementary Figures and Tables for "Bacterial virus lambda gpD-fusions to cathelicidins, α- and β-defensins, and disease-specific epitopes evaluated for antimicrobial toxicity and ability to support phage display."

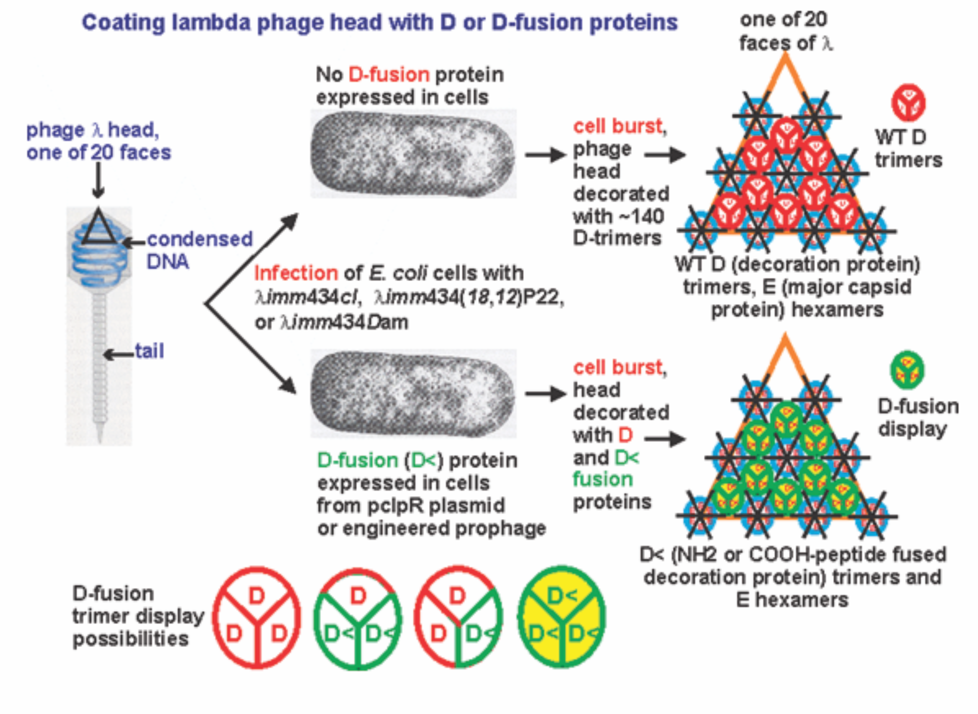

Figure S1. Coating lambda phage head with gpD or gpD-fusion proteins. The occurrence of the three gpD-fusion trimer display possibilities has yet to be determined (see text).

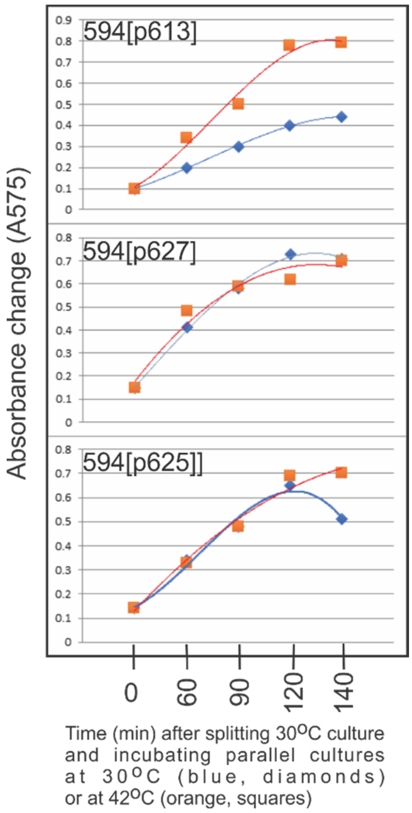

Figure S2. Comparison split culture growth after gpD, gpD-LL37 and gpD-PR39 expression. Comparison of culture growth at A_{575nm} after splitting 30°C cultures and incubating parallel cultures at 30°C and 42°C, where the expression of the plasmid encoded *D*, or *D*-fusion gene, respectively, is repressed, or induced. Comparison is shown for the *D*, or *D*-fusion genes in strains expressing *D*coe (plasmid p613), or strains expressing the *D*-fused cathelicidins: *D*coe-PR39 (plasmid p625), and *D*coe-LL37 (plasmid p627). For procedural information refer to Table 1 and 3 legend in text.

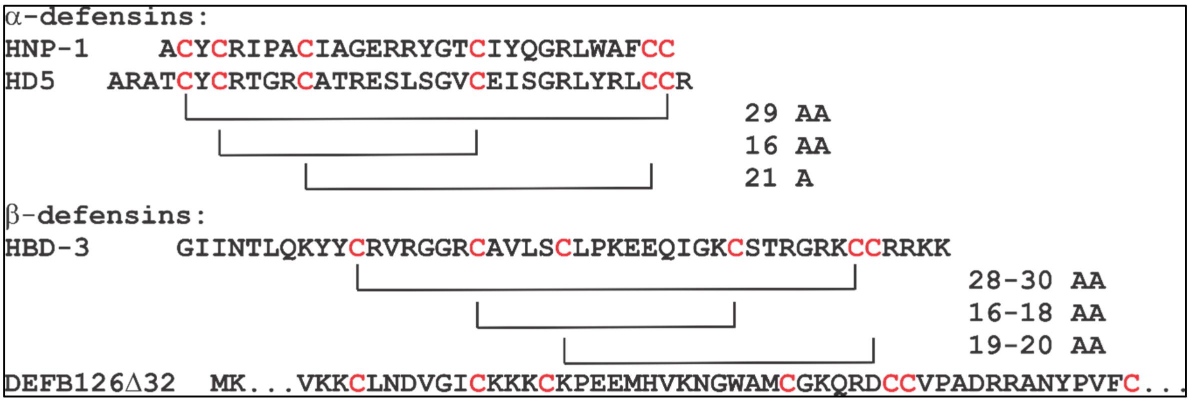

Figure S3. Predicted disulfide bonds formed in *D*-fusion defensins (41, 44, 45).

Table S1. Complementation by plasmid expression vectors of an amber mutation within the capsid decoration protein gene *D* in two lambdoid phages. Complementation was evaluated for plasmids that express three different *D*-fusion, or only gpDcoe. Each of the *D*-fusions include an 11 amino acid (GYMLGSAMSRP, termed YML) single epitope from the sequence of the cervid PrP protein (28). Plasmid p676 has NH_2-terminal gpD fusion; all other plasmids have COOH-terminal gpD fusions.

pcIpR-timm plasmid in host strain 594	EOP of infecting phage on cells with *D*-fusion plasmid						
	λ*cI*857 Dam123 (5.4x10^{8} pfu/ml)			λ*imm*434 Dam123 (4.9x10^{8} pfu/ml)			
	30°C	39°	41-42°	30°	37°	39°	41-42°
p675: pcIpR-Dwt-YML-timm	<10^{-5}	0.1	0.1	<10^{-5}	-	0.5	0.6
p676: pcIpR-MYML-Dcoe-timm	<10^{-5}	<10^{-5}	<10^{-5}	<10^{-5}	-	<0.1	≤0.1
p674: pcIpR-Dcoe-YML-tmm	<10^{-5}	0.3	0.5	<10^{-5}	-	0.7	0.9
p613: pcIpR-Dcoe-timm	-	-	-	<10^{-5}	0.8	-	0.8

Single-burst LDP-vaccine and SEV production. A few liters of λimm434*cI* lysate are prepared with a titer of >1x10^{10} pfu/mL. Cells transformed with a pcIpR-*D*-fusion-timm plasmid [USA and Canadian patents granted for "Platform genetic display system for phage display vaccine production"] that can express the *D*-fusion protein needed for preparing phage display particles, are isolated on LB-Amp50 agar plates. Single colonies are used to inoculate four two-liter flasks, each with 1 L LB-Amp50. The cultures are incubated with shaking to reach stationary phase, and the cells from each flask are

pelleted and resuspended in 1/10th volume of fresh LB medium (to about $1.5x10^{10}$ cfu/ml) and then incubated with shaking at 30°C for 30 min. The concentrated cells (derived from the initial two liters of culture cells) are then infected with *λimm*434*cI* at MOI of 3 to 5. The phage cell mixture is held at room temperature for 15 to 30 min without shaking. The infected cells are evenly transferred into four six-liter flasks, each with two liters of LB medium prewarmed to 39°C. Raising the cell temperature from 30 to 39°C permits expression of the *D*-fusion gene from the plasmids present in the cells. The culture absorbance is monitored at A_{575nm} for 60 to 90 min, and when it drops to about 0.05 the flasks are removed from the shaker, transferred to a cold room, the cells are pelleted, and the pellets discarded. The supernatant of the lysate is pooled in a carboy and solid polyethylene glycol (PEG, average molecular weight of 6000-7500) and NaCl are added to 4% and 0.5M, respectively, shaken into solution, and held overnight at 4°C. The lysate is spun at 8,000 revolutions per minute (rpm) for 15 to 20 minutes in a six-liter capacity JA 9.1000 Avanti rotor and the supernatant is discarded. The pellets are each resuspended overnight at 4°C in about 1 mL of buffer, adjusted to a refractive index of 1.382 with solid CsCl, and spun at 30,000 rpm in a Ti70 ultracentrifuge rotor (Coulter-Beckman) overnight at 4°C. The collected band(s) are pooled and re-banded in a Ti60 rotor. The banded phages are dialyzed prior to utilization for immunological assays against 200-1000X volume of phage dialysis buffer (0.3 to 0.5M NaCl, 0.01M Tris, 0.05M $MgCl_2$,pH 7.8), and then twice against 3000X PBS (0.0036 M KCl, 0.0014M KH_2PO_4, 0.136 M NaCl, 0.004M Na_2HPO_4, pH 7.4). *When only one liter of a lysate from each infection is needed*, as in experiments described in Figure 6, a scaled-down version of the single burst infection procedure is followed. The infection lysate is subjected to differential centrifugation: first at 8,000 rpm to remove the remaining nonlysed cells and large cell debris, and the subsequent lysate is spun in large polycarbonate tubes in a 60Ti rotor (Coulter-Beckman) for 24 hr at 30,000 rpm to pellet phage without introducing any treatment with PEG. The pellets are resuspended by covering them with 0.5 ml of-buffer made 0.01M in $MgCl_2$.buffer, allowing them to soften overnight at 4°C. The resuspended pellets are adjusted with solid CsCl to a refractive index of 1.382 and the phage are banded in an ultracentrifuge as described above. Any remaining free protein in the phage pellet is excluded and forms a thin band above the gradient.

Supplementary File S1:

Single epitope vaccines (SEV): a hypothesis and approach using phage display particles made from bacterial virus lambda.

Sidney Hayes

Department of Biochemistry, Microbiology & Immunology, College of Medicine, University of Saskatchewan, Saskatoon, SK S7N 5E5, Canada. sidney.hayes@usask.ca

Abstract: A hypothesis is advanced for the use of banked custom *E. coli* strains to prepare targeted rapid response vaccines comprising multiple single epitope bacterial virus (phage) *lambda display particles* (LDP). The LDP are formed during infection of a banked *E. coli* strain. The LDP do not encode the displayed polypeptide, which represents a fusion between the virus capsid decoration protein gpD and a foreign polypeptide. The high efficiency of displaying single epitope 17 amino-acid fusions to gpD suggests their potential use as single epitope vaccines (SEV).

Keywords: phage display; single epitope vaccines; phage lambda; gpD-fusion capsid display; banked vaccine paradigm

Peptide vaccines. There are many peptide vaccines being developed to viral infections and to cancers (1, 2). Synthetic peptides offer several advantages over other forms of vaccines, including rational engineering (computer aided) design (3) and safety factors related to reducing unnecessary antigenic load that is often present in vaccines that include whole organisms or large proteins (1). However, peptide vaccines on their own are often weakly immunogenic and require appropriate delivery systems to be effective. Examples of delivery methodology include emulsions, liposomes, producing polymeric particles, and their incorporation on virosomes. We describe a phage display system for vaccine production whereby peptide epitopes can be displayed at very high density on the capsid head of the bacterial virus (bacteriophage) lambda.

Bacteriophage as single epitope vaccine (SEV) display agents. Bacteriophages are bacterial viruses with genomes comprising single or double-stranded DNA, or single, or double-stranded RNA surrounded by a predominantly protein capsid that enables the viral particles to survive and thrive in diverse environmental conditions (4). Exploiting bacterial viruses as nanoparticles (5) has become a modern theme in biotechnology, *e.g.*, to find alternatives for antibiotics, as biocontrol agents, and as phage display vehicles for vaccines (6). Phage display particles have been used for vaccine delivery, *e.g.*, to deal with viral threat agents such as foot-and-mouth disease virus (7), porcine *Circovirus 2* (8), West Nile virus (9), HIV (10), classical swine fever virus (11), and to bacteria-spores-toxins (anthrax antigens) (12). All phage display strategies involve fusing the coding region for a protein or polypeptide to a phage surface capsid gene. The capsid-fusion gene is expressed from a phage genome engineered to include the gene fusion or is expressed from a surrogate genome. In our model, the lambda gene for its capsid decoration protein, gpD, is fused to genetic information for a protein or polypeptide. Display vaccines made via a surrogate gene expression system for the capsid fusion gene offers two advantages. First, the display particles generated do not need to be infective, and secondly, even if some remain infective, their infection of sensitive bacteria would yield the starting or wild type natural phage, thus preventing environmental dispersal/ propagation of the genetically engineered, potentially harmful variant, in turn, providing an element of safety against horizontal gene transfer.

Vaccine preparedness. A hypothesis is advanced for the use of banked custom *E. coli* strains to prepare targeted rapid response vaccines comprising multiple single epitope bacteriophage *lambda display particles* (LDP) that do not *encode* gene fusions to foreign peptides. The rationale and supporting LDP preparation criteria required for the system to work are described. This bacterial virus display-vaccine approach could generate phage-display vaccines in an hour or two following phage infection of host cells comprising preconstructed (banked) cell-plasmid fusion protein expression systems. When used experimentally, the system represents a methodology to permit rapid evaluation, *e.g.*, in mice, of diverse pathogenic peptide epitopes displayed on phage particles.

Hypothesis for SEV preparation

A vaccine made to an epitope representing an antigenically active, short, continuous region of a protein is termed a *single epitope vaccine*, SEV. Described below is a hypothesis for generating polyclonal single epitope vaccines involving the use of banked, custom *E. coli* strains (in present iteration). This technology involves assembling "banked" *E. coli* bacteria with custom designed expression systems that will each produce a unique display polypeptide. Infecting one or more strains simultaneously, e.g. three such strains, from the cell bank with a harmless bacteriophage produces a trivalent vaccine. The vaccine results from high level production of the fusion polypeptide (one per valency per strain) and coated phage-particles from each strain infected. We have shown that both the cells expressing the gene fusion and the coated phage particles bursting from the infected cells induce mucosal immune responses when taken up by Peyer's patches in the small intestine (13). We suggest the following single epitope vaccine (SEV) formulation hypothesis (parts a-g) and prediction g1:

a) Relatively conserved regions of antigenically active segments of proteins can be displayed as disease-specific epitopes for vaccine candidate pools.

b) Vaccines made to a single epitope, or collections of SEV to several conserved antigenically active segments of one or more conserved proteins can be protective.

c) Synthetically generated genetic copies of antigenically active regions of pathogenic proteins (both natural or purposely randomized) can be manipulated by converting the information into a gene fusion which is then transformed into a harmless laboratory strain of bacterium (as *Escherichia coli*) and the transformed cells are then banked (stored freeze dried or frozen) as a rapid response repository for easily testing their utility and versatility to make protective SEV.

d) When needed, the conditionally expressible synthetic gene fusions, each representing a single disease-specific epitope, can be generated for each member of the collection of banked, transformed harmless bacteria.

e) SEV to each of the epitopes can be generated by phage display, permitting near-fully coated (very high display density) particles that are non-infectious to mammals, and which do not genetically encode the pathogenic epitope.

e1) Biosafety-epitopic DNA containment: Phage display particles (DP), where only the capsid is coated with the protein comprising the natural capsid component fused to the single display epitope, are strongly preferred to phage particles derived from phages where the gene fusion was genetically engineered into the phage genome. [Any escape of the DP resulting in the infection of a susceptible bacterium will only generate the harmless natural phage. In contrast, the escape of an engineered phage will disseminate DNA copies of the genetically engineered epitope(s) into the environment, which can provide a repository for potential horizontal gene transfer and dissemination of DNA encoding an epitope derived from a pathogen.]

e2) A measure of intellectual property protection: The SEV cannot be directly used to make more of it. For example, even if some vaccine LDP remain viable (assuming a replication defect is not engineered into it), their purification and regrowth on a sensitive bacterial host will only generate the display vector sequence devoid of the displayed epitope. Characterization of a SEV LDP will require possession of the banked custom *E. coli* strain, purification and fractionation of the LDP, and use of something like Q-Tof mass spectroscopy to describe the fusion peptide linked to the capsid display protein.

e3) In a single reaction, multiple SEV display phage candidates can be produced by simultaneously co-infecting multiple pooled banked strains with a single display phage precursor.

f) Both the cells induced to express the synthetic gene fusion representing the single disease-specific epitope and the phage display particles (PDP) grown up on these cells can serve (or serve together) as a mucosal SEV, and each are taken up in Peyer's patches within the intestine and each stimulate both cellular and T-cell mediated immune responses. Various means for killing the transformed, banked, bacterial cells are possible, or can be engineered, if essential.

g) The banked bacterial strains, each capable of expressing gene fusions of a single pathogenic epitope permit the generation of rapid response antimicrobial vaccines (or, additionally, to potentially generate agents able to neutralize existing or synthetic toxins, or for production of synthetic antimicrobials). This represents a new paradigm for generating universal vaccines, or for responding rapidly to unaccounted infectious or potential pandemic agents, or possibly to other biohazards.

g1) Vaccine Formulation Prediction: Single dose vaccines can be made up, as needed using pre-designed "banked" *E. coli* bacteria with custom designed expression systems that can each produce a unique display polypeptide. (More complex and effective versions of what follows are no doubt possible with empirical experimentation.) Our strategy: A small screw-capped plastic bottle, e.g., the size of a small commercial pill bottle, can be designed that has three sealed compartments, separated by foil or a thin plastic layer, which can each be perforated, e.g. by a pencil, pen, or attached sterile plastic stick. In one compartment (lower) are freeze-dried cells that have been banked, representing a single strain for preparing a single SEV or multiple banked strains for preparing multiple SEVs in one reaction. In another compartment are freeze-dried (or in some way suspended) phage particles (or perhaps the freeze-dried cells and freeze-dried phages can be mixed together and placed in a single compartment). In a third (or second) compartment is contained sterile growth medium that

will permit both cell growth and phage infection resulting in a burst of phage display particles representing one, or several, SEV types. Once the separating layers are perforated and the container is shaken and incubated for a few hours (on the candidate's body at 37°C) the contents can be drunk. (Alternatively, a gentle method for killing the cells could be introduced before ingestion.) The ingested cells and DP will be taken up in Peyer's patches within the gut resulting in humeral and cellular mucosal immune responses. [Is this such a radical idea, considering that many consume millions of live probiotic microorganisms daily in various yogurt combinations? See (14).]

Our initial attempt at preparing phage display vaccines devolved from an expectation that simultaneously displaying multiple epitopes, i.e., several discontinuous portions of a pathogenic protein would produce a broad immune response to that protein. This approach for rationale design of epitope-based vaccines has been termed "string-of beads" vaccines (15). In the companion manuscript, we have shown that 17 amino acid COOH-terminal additions to the capsid protein gpD can efficiently substitute for inactive *D* on an infecting lambda phage. Importantly, *enough* expression of the *D*-fusion arises from the plasmid expression system within cells growing at 37°C to permit highly efficient suppression of a defect in *D*. This would support the vaccine formulation prediction, g1 of the single epitope vaccine formulation hypothesis, permitting phage lysate formation to occur while held during the period of cell infection on the person to receive it. The short-term value of this scheme may be to represent a simple procedure, in combination with mouse or small animal bioassays, for evaluating the immunogenic/ protective activity of continuous single epitopes derived from pathogen proteins.

Advantages of the SEV disease therapy paradigm

The technology involves assembling "banked" *E. coli* bacteria with custom designed expression systems that will produce a unique display polypeptide. Infecting a custom-made-bacteria from the bank with a harmless bacterial virus results in high level production of the vaccine or antimicrobial agent in the form of a coated phage particle. There is no need to stockpile doses of many types of vaccines, or antimicrobial or toxin-enveloping particles. The ability to draw upon an inexpensive bank of custom-designed bacterial strains from which phage display vaccines, antimicrobial agents, or toxin-enveloping particles can be made on a moment's notice is a new disease therapy paradigm. Modern genetic technology enables the synthetic design and production of a virtually limitless array of vaccine or antimicrobial phage display particles, where each particle can be rapidly made to high dosage quantities using a platform genetic technology patented in the USA (patent no. 8,663,913 B2) and Canada (patent no. 2,761,105).

Besides rapid production of SEV's, there is no cold chain, so that refrigerated storage, or even the storage of vaccine doses, becomes unnecessary. The methodology was suggested in the vaccine formulation prediction, g1.

Banked *E. coli* strains capable of producing any engineered epitope that is imagined can be used to produce SEV for evaluating the utility (immunogenicity/ protective activity) of the given epitope to a threat agent, using small animal bioassays.

Potential Examples for SEV

A few examples illustrate the potential of the SEV hypothesis using synthetic biology: Our proposal would be directly applicable for displaying any of the multivalent peptides identified as potential vaccine candidates to multiple types of human papillomavirus (3). Alternatively, the influenza A virus extracellular domain Matrix 2 viroporin protein contains 97 amino acids, is expressed from spliced mRNA, and plays an important role during the early stages of virus entry. It can be divided into three parts, the extracellular N-terminal domain (Me2 positions 2-24), the transmembrane (TM) domain (positions 25-46) and the intracellular C-terminal domain (positions 47-97). The M2e domain represents a promising epitope for designing a universal vaccine. The N-terminal 9 amino acids are almost absolutely conserved; whereas, M2e residues 10 to 24 are more variable, even though some of these residues are strongly conserved. [Figure 1 in (16) shows consensus M2e sequences based on 14,588 human, 9324 avian and 3060 swine M2 sequences.] For

example, one could display *D*-fusions for M2e amino acids 10-20 to human A (PIRNEWGCRCN), avian A (PTRNEWECRCS), and swine A (PIRNGWECKCN) (17). Other synthetic versions of these epitopes modified at the variable amino acid sites could be constructed and banked for future challenge evaluation. Relative to the SEV hypothesis, the simultaneous infection by λ*imm*434D^- phage into three banked host cells, each expressing *D*-fused to one of the 11 amino acid M2e variations, can produce three high density phage-display vaccine preparations to human, avian and swine variants of M2e in one short infection cycle. M2e has been coupled to a plethora of carriers to enhance anti-M2e immune responses in vaccination experiments. The degree of M2e epitope density on the carrier molecule is a critical factor for the induction of a strong M2e-specific immune response [reviewed in (16)].

Another synthetic engineering possibility might be to produce vaccine candidates to a highly toxic protein for which no antidote exists, *e.g.,* the newly discovered *Clostridium botulinum* type H toxin (18), without isolating the toxin or conversion of the protein to a toxoid. Synthetic *D*-fusion display technology could help evaluate for potential neutralizing vaccine epitopes, based upon comparative DNA sequence analysis. Indeed, using sequence comparisons, it may prove possible to discern universal epitopic regions of interest for neutralizing the A through H toxins (19).

Summary

We suggest the stockpiling of banked custom *E. coli* strains, that include pre-designed *D*-fusion expression plasmids, for preparing targeted, rapid-response vaccines and vaccine evaluation tools. Many disease specific epitopes (DSE) are already recognized among harmful microbes. Infecting bacteria from a stored cell bank with a harmless bacterial virus can generate high level production of the vaccine in the form of coated phage particles. One dose, as described in the SEV hypothesis could produce particles of an equivalent concentration to the level used to vaccinate pigs to *Circovirus 2* (20). This represents a new paradigm for rapidly scaling up and responding to health emergencies dealing with pathogens. The long lag required for vaccine preparation to potential pathogen unknowns requires greater attention to novel vaccine strategies.

References

1. Li W, Joshi MD, Singhania S, Ramsey KH, Murthy AK. Peptide Vaccine: Progress and Challenges. Vaccines (Basel). 2014;2(3):515-36.
2. Purcell AW, McCluskey J, Rossjohn J. More than one reason to rethink the use of peptides in vaccine design. Nat Rev Drug Discov. 2007;6(5):404-14.
3. Dey S, De A, Nandy A. Rational Design of Peptide Vaccines Against Multiple Types of Human Papillomavirus. Cancer Inform. 2016;15(Suppl 1):1-16.
4. Jonczyk E, Klak M, Miedzybrodzki R, Gorski A. The influence of external factors on bacteriophages--review. Folia Microbiol (Praha). 2011;56(3):191-200.
5. Namdee K, Khongkow M, Boonrungsiman S, Nittayasut N, Asavarut P, Temisak S, et al. Thermoresponsive Bacteriophage Nanocarrier as a Gene Delivery Vector Targeted to the Gastrointestinal Tract. Mol Ther Nucleic Acids. 2018;12:33-44.
6. Haq IU, Chaudhry WN, Akhtar MN, Andleeb S, Qadri I. Bacteriophages and their implications on future biotechnology: a review. Virol J. 2012;9:9.
7. Ren ZJ, Tian CJ, Zhu QS, Zhao MY, Xin AG, Nie WX, et al. Orally delivered foot-and-mouth disease virus capsid protomer vaccine displayed on T4 bacteriophage surface: 100% protection from potency challenge in mice. Vaccine. 2008;26(11):1471-81.
8. Hayes S, Gamage LN, Hayes C. Dual expression system for assembling phage lambda display particle (LDP) vaccine to porcine Circovirus 2 (PCV2). Vaccine. 2010;28(41):6789-99.
9. Herrmann S, Leshem B, Lobel L, Bin H, Mendelson E, Ben-Nathan D, et al. T7 phage display of Ep15 peptide for the detection of WNV IgG. J Virol Methods. 2007;141(2):133-40.
10. Sathaliyawala T, Rao M, Maclean DM, Birx DL, Alving CR, Rao VB. Assembly of human immunodeficiency virus (HIV) antigens on bacteriophage T4: a novel in vitro approach to construct multicomponent HIV vaccines. J Virol. 2006;80(15):7688-98.

11. Wu J, Tu C, Yu X, Zhang M, Zhang N, Zhao M, et al. Bacteriophage T4 nanoparticle capsid surface SOC and HOC bipartite display with enhanced classical swine fever virus immunogenicity: a powerful immunological approach. J Virol Methods. 2007;139(1):50-60.
12. Shivachandra SB, Rao M, Janosi L, Sathaliyawala T, Matyas GR, Alving CR, et al. In vitro binding of anthrax protective antigen on bacteriophage T4 capsid surface through Hoc-capsid interactions: a strategy for efficient display of large full-length proteins. Virology. 2006;345(1):190-8.
13. Gonzalez-Cano P, Gamage LNA, Marciniuk K, Hayes C, Napper S, Hayes S, et al. Lambda display phage as a mucosal vaccine delivery vehicle for peptide antigens. Vaccine. 2017;35(52):7256-63.
14. Sarhan WA, Azzazy HM. Phage approved in food, why not as a therapeutic? Expert Rev Anti Infect Ther. 2015;13(1):91-101.
15. Schubert B, Kohlbacher O. Designing string-of-beads vaccines with optimal spacers. Genome Med. 2016;8(1):9.
16. Deng L, Cho KJ, Fiers W, Saelens X. M2e-Based Universal Influenza A Vaccines. Vaccines (Basel). 2015;3(1):105-36.
17. Liu W, Zou P, Ding J, Lu Y, Chen YH. Sequence comparison between the extracellular domain of M2 protein human and avian influenza A virus provides new information for bivalent influenza vaccine design. Microbes Infect. 2005;7(2):171-7.
18. Barash JR, Arnon SS. A novel strain of Clostridium botulinum that produces type B and type H botulinum toxins. J Infect Dis. 2014;209(2):183-91.
19. Dover N, Barash JR, Hill KK, Xie G, Arnon SS. Molecular characterization of a novel botulinum neurotoxin type H gene. J Infect Dis. 2014;209(2):192-202.
20. Gamage LN, Ellis J, Hayes S. Immunogenicity of bacteriophage lambda particles displaying porcine Circovirus 2 (PCV2) capsid protein epitopes. Vaccine. 2009;27(47):6595-604.
